# Simultaneous prediction of RNA secondary structure and helix coaxial stacking

**DOI:** 10.1186/1471-2164-13-S3-S7

**Published:** 2012-06-11

**Authors:** Pooya Shareghi, Yingfeng Wang, Russell Malmberg, Liming Cai

**Affiliations:** 1 Department of Computer Science, University of Georgia, Athens, GA 30602, USA; 2 Institute of Bioinformatics, University of Georgia, Athens, GA 30602, USA; 3 Department of Plant Biology, University of Georgia, Athens, GA 30602, USA

## Abstract

**Background:**

RNA secondary structure plays a scaffolding role for RNA tertiary conformation. Accurate secondary structure prediction can not only identify double-stranded helices and single stranded-loops but also help provide information for potential tertiary interaction motifs critical to the 3D conformation. The average accuracy in *ab initio *prediction remains 70%; performance improvement has only been limited to short RNA sequences. The prediction of tertiary interaction motifs is difficult without multiple, related sequences that are usually not available. This paper presents research that aims to improve the secondary structure prediction performance and to develop a capability to predict coaxial stacking between helices. Coaxial stacking positions two helices on the same axis, a tertiary motif present in almost all junctions that account for a high percentage of RNA tertiary structures.

**Results:**

This research identified energetic rules for coaxial stacks and geometric constraints on stack combinations, which were applied to developing an efficient dynamic programming application for simultaneous prediction of secondary structure and coaxial stacking. Results on a number of non-coding RNA data sets, of short and moderately long lengths, show a performance improvement (specially on tRNAs) for secondary structure prediction when compared with existing methods. The program also demonstrates a capability for prediction of coaxial stacking.

**Conclusions:**

The significant leap of performance on tRNAs demonstrated in this work suggests that a breakthrough to a higher performance in RNA secondary structure prediction may lie in understanding contributions from tertiary motifs critical to the structure, as such information can be used to constrain geometrically as well as energetically the space of RNA secondary structure.

## Introduction

RNA secondary structure plays the critical role of scaffolding the tertiary structure (i.e., 3D conformation) [1-5]. In the secondary structure, Watson-Crick (AU and GC) and wobble GU pairs form double-stranded helices that enclose unpaired, single-strand loops [6]. The distinguishable pattern of canonical base pairs has enabled *ab initio *prediction of the secondary structure, typically by minimization of the global free energy associated with involved structure elements [7-10]. In the past three decades, considerable success has been made in secondary structure prediction, e.g., with average accuracy of about 70% [11-13], and offered a viable venue toward RNA tertiary structure prediction [4,5,13,14]. However, prediction performance breakthroughs have been limited to short RNA sequences; improvements on the accuracy for longer RNA sequences have relied on multiple related sequences [15,16], which are often not available, or profile based alignments [17,18], which can only be effective for known structures.

Elements of the secondary structure are interrelated with tertiary interaction motifs [2,4,19], which consist of less understood non-canonical base pairs, with some just being revealed recently [19,20]. Such motifs bundle and connect helices to form and stabilize the tertiary structure. As a common local motif, two helices sharing a contiguous backbone strand may coaxially stack resulting in an energetically more stable pseudo-contiguous helix [21,22]. Coaxial helices are prevalent in known RNA tertiary structures, for instance accounting for 32% of 613 tertiary interactions in 54 high-resolution RNA structures investigated by Schlick group [23]. In particular, they are present at about 84% of multiple loop junctions involved in these structures. Since junctions are single-strand loops joined and enclosed by helices, computational methods effective on prediction of coaxial stacking would substantially improve the performance of the secondary structure prediction as well.

There were only a few previous results in computational investigation of RNA helix coaxial stacking. Walter *et al *[24] demonstrated in a case study that base-pair to base-pair stacks between terminal base pairs of two neighboring helices provide free energy improvement for the predicted secondary structure. Tyagi and Mathews [22] tested the idea of predicting coaxial stacking by free energy minimization using nearest-neighborhood parameters on known RNA secondary structures. They showed the potential to predict coaxial stack with free energy minimization when the number of intervening mismatches between stacked helices is small. In the comparative analysis of 3-way junctions joined by three helices, Lescoute and Westhof measured distance distributions between the two coaxially stacked helices within the junctions [25]. For junctions of four-ways and of higher orders, it was observed by Schlick group [26,27] that coaxial stacking occurs preferentially in helices adjacent to loops of small size and rich in adenine. In this paper, we present a new method for the prediction of RNA secondary structure and coaxial stacking. Different from previous secondary structure prediction methods, ours can produce information of coaxially stacked helices included in the predicted structure. Unlike prediction of coaxial stacking upon an already predicted secondary structure, the new method offers the simultaneous prediction of the two. We discovered and applied rules of coaxial stacking, including both sequential and structural patterns, to the prediction of secondary structure. Such rules constrain possible energetic and geometric relationships between helices to be predicted, resulting in a reduced space of alternative structures and a potential improvement in secondary structure prediction.

The new method has been developed into a dynamic programming application (called RNAcoast). We conducted tests on five families of ncRNAs, of total 386 sequences, to evaluate the capability of the new method in simultaneous prediction of secondary structure and coaxial stacking. These ncRNAs were retrieved from Rfam database [28], of short to moderately long lengths, with and without coaxial stacking in the tertiary structure. RNAcoast produced comparable predictions as the state-of-the-art program RNAfold on all cases, it outperformed the latter on tRNAs, where coaxial stacks are present, by an additional 17% accuracy, a significant leap from the average performance (i.e., 60-70% of the number of correct base pairs) achievable by previous energy based models on tRNAs. The test results demonstrate that coaxial stacking rules can successfully narrow down a possibly large number of alternative structures within 5-10% of the predicted minimum energy, which would otherwise be difficult to distinguish.

## Results

We implemented the algorithm into a program named RNAcoast. We tested five ncRNA sets of sequences on our program and compared the predicted secondary structure with the original Rfam annotation to evaluate its accuracy. We also tested these ncRNA sequences on the state-of-the-art secondary structure prediction program RNAfold [9,29], and made performance comparisons between the mentioned programs. All test data and results are available at: http://www.cs.uga.edu/~shareghi/RNAcoast.

### Data preparation

We downloaded five ncRNA datasets from seed alignments of Rfam. Ninety-five (10% of) tRNA sequences were randomly picked up from the corresponding seed alignment of 967 tRNAs. All ninety-eight available Intron Group II sequences and all eighty-four available Hammerhead type III sequences were retrieved directly from seed datasets. We also downloaded all 30 Intron Group I sequences available from its seed alignment, and extracted the P4P6 domain of each sequence. Similarly, we retrieved all 79 HCV IRES sequences available from its seed alignment, and extracted domain III of each sequence. The average lengths of tRNAs, Intron group II, Hammerhead type III, P4P6, and domain III of HCV IRES are 73.62, 87.18, 55.36, 126, and 111.68, respectively. Many of these sequences contain long inserted regions compared to their annotated consensus structures, with lengths greatly exceeding the corresponding average lengths (see Table 1). Therefore, these collections of ncRNA sequences cover short to moderately long lengths. Coaxial stacks are present in tRNAs, Hammerhead type III, and HCV IRES domain III, while they are not present in the consensus of Intron Group II or the consensus of P4P6 domain. However, some P4P6 sequences have a long insertion region containing a three-way junction, where coaxial stacking may occur. The secondary structures of these ncRNAs vary as well, from simpler structures in Hammerhead type III and Intron Group II to more sophisticated tRNAs and some P4P6 sequences containing the inserted 3-way junction and a GAAA tetra-loop [1].

**Table 1:** Sensitivity based on the number of correctly predicted base pairs

ncRNA	Num. of sequences	**Avg len**.	**Min len**.	**Max len**.	**Sensitivity (****RNAcoast****)**	**Sensitivity (****RNAfold****)**
**Hh3**	84	55	40	82	85.04%	95.71%
**tRNA**	95	74	66	93	81.67%	64.59%
**Intron-gII**	98	87	42	154	81.94%	83.71%
**P4P6**	30	126	58	191	57.42%	64.62%
**HCV**	79	112	85	116	83.01%	78.43%

Performance comparison between RNAcoast and RNAfold measured by the number of correctly predicted base pairs.

### Performance in secondary structure prediction

We conducted two types of evaluations on the predicted structures. One is to consider the percentage of base pairs correctly predicted by the programs. The other is to consider the number of sequences whose overall structure topology is correctly predicted. Shown in the next section, we also evaluated the capability of RNAcoast in predicting coaxial stacks.

Table 1 summarizes the performance of RNAcoast vs RNAfold with reference to the original annotated consensus structures for the tested ncRNAs. The sensitivity is computed as

$$\text{Sensitivity}=\frac{TP}{TP+FN}\times 100\%$$

where *TP *is the number of true positives (i.e. correctly predicted base pairs) and *FN *the number of false negatives (i.e. missed base pairs). The results show that for short sequences of simpler secondary structures, i.e., Hammerhead type III and Intron Group II, both RNAcoast and RNAfold performed well, with RNAcoast slightly less accurate than RNAfold. Also, for longer sequences in HCV IRES domain III dataset, both programs performed well, with RNAcoast slightly more accurate than RNAfold.

Test results on the tRNA data set demonstrates the true advantage of incorporating coaxial stacking into prediction of ncRNAs that may contain coaxial stacking motifs. RNAcoast outperformed RNAfold by more than 17% accuracy, a significant leap from the average performance (i.e., 60-70%) achievable by previous energy based models on tRNAs. The coaxial stacking rules successfully narrowed down a possibly large number of alternative structures within 5-10% of the predicted minimum energy, which would otherwise be difficult to distinguish [12].

**Figure 1:**
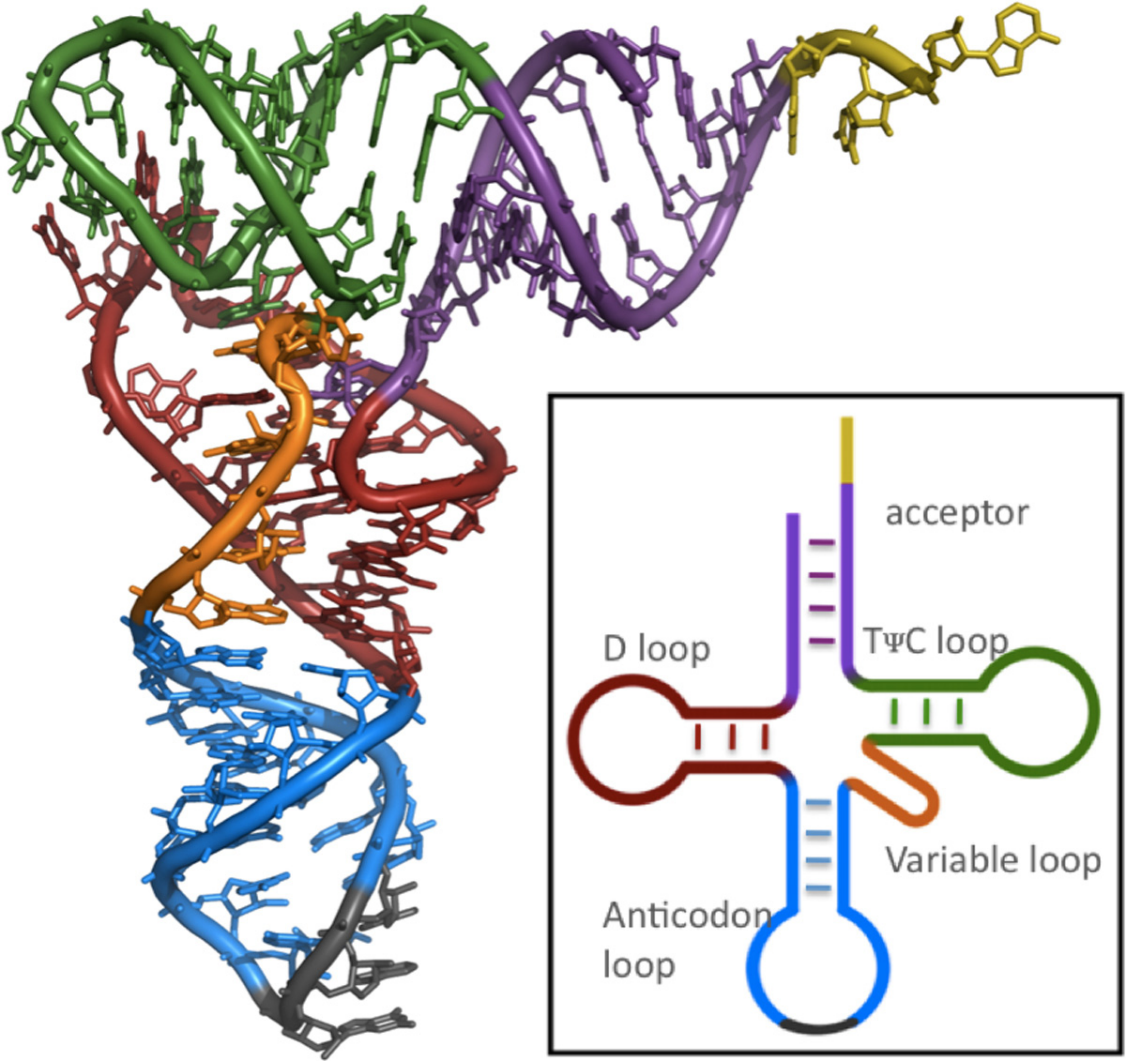
**The general tRNA tertiary structure (and the secondary structure in the box)**. Four helices (in acceptor, *D*-arm, *T*_Φ_*C *arm, and anticodon arm) enclose loops, including the variable loop (orange), possibly long in some tRNAs. The helix of acceptor (purple) and the helix of *T*_Φ_*C *arm (green) coaxially stack (in the nested fashion); the helix of *D*-arm (red) and the helix of anticodon arm (blue) coaxially stack (in the parallel fashion). Figure modified from Rfam [28].

Table 2 shows that the performance of RNAcoast was actually even better when the real structure of these tRNA sequences were examined against the consensus. RNAcoast captured the secondary structure topology correctly for more than 72% of sequences. We carefully examined those sequences whose topologies were not predicted correctly and were able to identify that half of them actually have a long variable loop (see Figure 1) which contains an extra helix, some correctly predicted by RNAcoast and RNAfold. Therefore, the percentage of correctly predicted topologies for RNAcoast was actually 86%, consistent with the sensitivity calculated based on correctly predicted base pairs. Since these tRNAs were randomly sampled from 967 sequences of the seed alignment in Rfam, the test results demonstrate the effectiveness of our method.

**Table 2:** Sensitivity based on the number of correctly predicted topologies

ncRNA	Topology sen. (%)		Adjusted topology sen. (%)	
	
	**RNAcoast**	**RNAfold**	**RNAcoast**	**RNAfold**
**Hh3**	75	92.86	N/A	N/A
**tRNA**	72.63	24.21	86.32	27.37
**Intron-gII**	75.51	84.69	N/A	N/A
**P4P6**	30	56.67	66.67	86.67
**HCV**	74.68	75.95	N/A	N/A

Performance comparison between RNAcoast and RNAfold measured by the number of correctly predicted secondary structures.

We point out that the relatively low sensitivity for RNAcoast on Hammerhead ribozyme type III shown in Table 2 was due to the extra stem-loop it predicted within the three-way junction, much as the situation of the variable loop of tRNAs.

For longer sequences of P4P6, counting correctly predicted base pairs appeared to distance RNAcoast a little more from RNAfold; but neither programs achieved a satisfactory sensitivity. The underperformance may be explained by the nature of the P4P6 sequences and the reference consensus structure from Rfam. Out of the thirty sequences tested, 12 of them have lengths exceeding 150 (but under 191), 6 sequences have lengths below 90, and another 12 have lengths in between. The consensus structure from Rfam was based on the smallest group of short sequences, leaving a long inserted region for others. Though both programs were able to predict the substructure formed in the inserted region, but the small number of base pairs annotated in the consensus made them easy to be missed by both programs. However, in spite of the low number of base pairs correctly predicted by RNAcoast, the program was able to achieve an adjusted 67% sensitivity in topology prediction.

### Performance in coaxial stacking prediction

To evaluate the performance of our method in coaxial stacking prediction, we computed both the sensitivity and positive predictive value (PPV) on the number of correctly predicted coaxial stacks. The PPV is defined as

$$\text{PPV}=\frac{TP}{TP+FP}\times 100\%$$

where *FP *stands for false positive, the number of incorrectly predicted coaxial stacks.

Table 3 shows both PPV and sensitivity for RNAcoast to predict coaxial stacks on tRNA, Hammerhead Type III, and HCV IRES domain III sequences, where coaxial stacks are present. There were 190 coaxial stacks in the 95 tRNAs, with two for each, 84 coaxial stacks in Hammerhead type III, with one in each, and 158 coaxial stacks in HCV, with two for each. The program was more specific on tRNAs, achieving a PPV of 75%, compared to 61% on Hammerhead type III, and 66% on HCV IRES domain III. It had the lowest sensitivity, 47%, on HCV IRES domain III compared with the other two families where sensitivity was around 70%.

**Table 3:** PPV and sensitivity based on the number of correctly predicted coaxial stackings

ncRNA	Num of sequences	TP	FP	PPV(%)	Sensitivity(%)
**tRNA**	95	130	44	74.71	68.42
**Hh3**	84	59	37	61.45	70.23
**HCV**	79	74	38	66.07	46.83

Performance of RNAcoast in prediction of coaxial stackings.

We compare these results with a previous work by Tyagi and Mathews who tested the idea of coaxial stack prediction using the energy minimization with nearest-neighbor parameters [22] on 31 ncRNAs (with known secondary structures and crystal tertiary structures). We notice that there were 17 tRNA sequences among these 31 sequences, for which the average PPV and sensitivity reported in the literature [22] were 58% and 66%, respectively on *k *= 0 and *k *= 1, where *k *is the number of unpaired nucleotides at the point of backbone joining of the two coaxially stacked helices.

## Discussion

While our program, RNAcoast, produced comparable predictions as the state-of-the-art program RNAfold on all cases, it outperformed the latter on tRNAs, where coaxial stacks are present, by more than 17% accuracy, a significant leap from the average performance (i.e., 60-70% of the number of correct base pairs) achievable by previous energy based models on tRNAs. Furthermore, RNAcoast predicted 86% of secondary structure topologies correctly, while RNAfold only predicted 27% of topologies correctly. Our coaxial stacking rules can successfully pick out the most plausible one from a possibly large number of alternative structures within 5-10% of the predicted minimum energy, which would otherwise be difficult to distinguish [12]. Such a performance is encouraging to solving the problem of RNA tertiary structure prediction.

We point out the small differences in performance between RNAcoast and RNAfold on Hammerhead type III and Intron Group II were most likely due to the simple strategy to exclude loop energies which was built into the current version RNAcoast. This was a little more of an issue for long sequences in the P4P6 dataset, which became serious when the consensus structure did not include a long inserted region. While improving the performance of RNAcoast can be achieved by incorporating the dismissed loop energies, a strategy different from evaluating predictions against the consensus structure may help as well.

We did not use the positive predictive value (PPV) to measure the performance in the correctly predicted base pairs. This was because some base pairs not belonging to the consensus structure but predicted by the programs may be valid if they fall in inserted regions of the consensus structure. Counting such base pairs as false positives would be bias against sequences substantially longer than the consensus. The situation was evident by our tests on these sequences, typically tRNAs where the variable loop may contain an extra stem-loop.

We have also examined the coaxial stacking prediction on the P4P6 sequences by RNAcoast. In contrast to RNAfold that predicted 8 three-way junctions in the long inserted region, our program predicted 5 three-way junctions in that region, with the same left nested coaxial stack predicted for 3 out of the 5 three-way junctions. Such a predicted coaxial stack has yet to be verified as it was counted as a real motif in one work [25] while was not by another [22].

The outcome of the tests on tRNAs is most interesting. The secondary structures of tRNAs were difficult to predict from individual sequences with energy-based methods, in spite of the conserved native structure across types and species. This is because a tRNA may have many alternative structures with free energies within 5-10% of the minimum free energy.

## Conclusions

This work introduced a new method for simultaneous prediction of RNA secondary structure and coaxial stacking between helices. The aim of the incorporation of coaxial stacking detection included improving the performance of energy-based *ab initio *secondary structure prediction. Our research identified sequential, energetic, and geometric rules for helix coaxial stacking to apply to a dynamic programming algorithm for secondary structure prediction. Results from testing the implemented program RNAcoast on five ncRNA datasets obtained from Rfam demonstrated the effectiveness of our method.

The significant leap of performance on tRNAs in this work suggests that a breakthrough to a higher performance in RNA secondary structure prediction may lie in understanding contributions from tertiary motifs critical to the structure, as such information can be used to constrain geometrically as well as energetically the space of RNA secondary structure. Since coaxial stacking is still a local tertiary motif, incorporating information of tertiary motifs of higher orders, such junctions, may further improve the prediction performance.

## Methods

**Figure 2:**
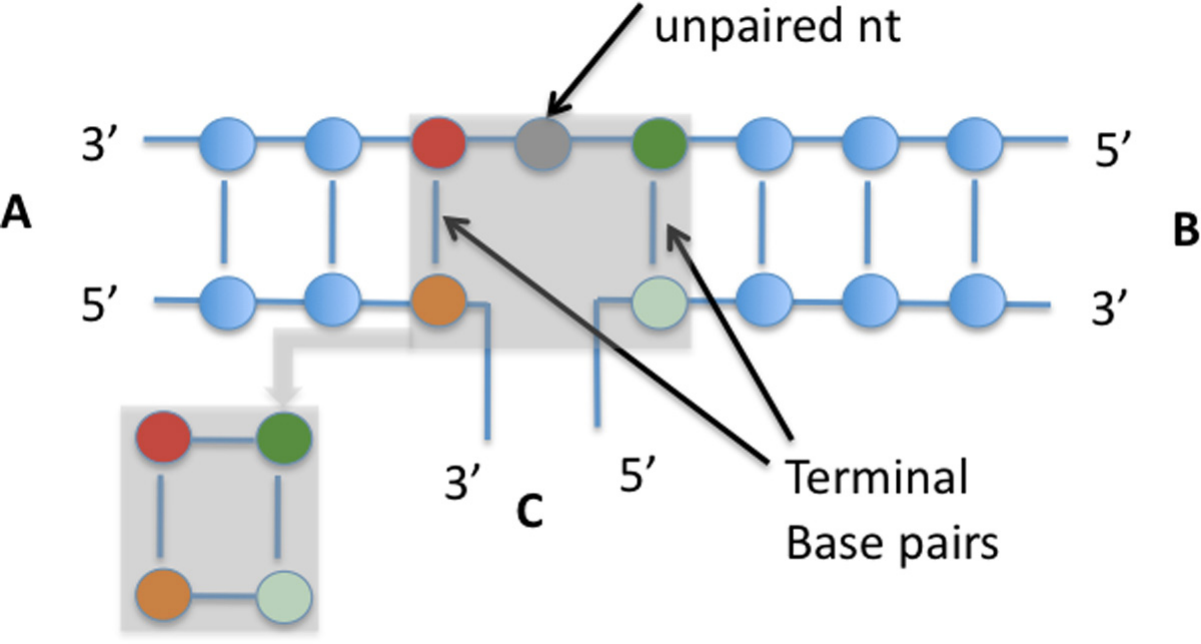
**Coaxial stacking of helices**. A secondary structure illustration of a coaxial stacking between two helices that share the same contiguous single-strand loop, in which unpaired nucleotides may be present. The terminal base pairs from both helices stack each other, resulting in an extra energy reduction calculated as if they were contiguous base pairs (shown in the callout). *A, B, C *represent the three substructures connected to the two helices, where exactly two substructures can be formed each by one contiguous backbone, and exactly one substructure by two separate backbones. If substructure *A *or *B *is formed by two separated backbones, the coaxial stacking is nested; if *C *is formed by two separated backbones, the stacking is parallel.

In the secondary structure, canonical base pairs form double-stranded stems (called helices in tertiary structure) that join and enclose unpaired, single-strand loops. Figure 1 shows the secondary and tertiary structure of tRNAs in general, which consist of four helices enclosing loops. Two neighboring helices joined by a contiguous single-strand loop *coaxially stack *if they share the same axis in the tertiary structure with the two terminal base pairs of respective helices stacking on each other at the joining point. Figure 2 gives a schematic illustration of two coaxially stacked helices. Depending on where the 5' and 3' ends of the sequence are connected to, the coaxial stack can be *nested *or *parallel *(see definition in the section below). Figure 1 shows two pairs of coaxial stacks in the four-way junction of the tRNA tertiary structure. We introduce a new method for simultaneous prediction of RNA secondary structure and coaxial stacks. Our strategy is to reward each potential coaxial stacking with the amount of negative energy incurred by the stacking and to incorporate both the energetic and geometric rules into the secondary structure prediction process. The energy of coaxial stacking is calculated as that contributed by the two stacked terminal base pairs of the coaxial helices (see Figure 2). This is an approximate quantity as the full mechanism for coaxial stacking to stabilize the involved structure is still to be fully understood due to additional tertiary interactions often detected at multi-way junctions where coaxial stacking usually occurs.

### Coaxial stacking rules

Previous investigations on three-way junctions [25] and junctions of higher orders [22,26,27] have revealed the small number *k *of unpaired nucleotides present at the joining loop between the two helices involved in a coaxial stacking. To verify this phenomenon for a wider spectrum of ncRNAs, we conducted a survey on the 51 sets of ncRNA seed alignments from Rfam [28], which had been used by software Infernal [18] as benchmarks. We computed the thermodynamic free energy of every helix instance using the RNAeval component of the Vienna RNA Package [9,29].

Based on this survey, we were able to identify two energy thresholds: less than -2.5 Kcal/mol for *semi-stable helices*, and less than -3.7 Kcal/mol for *stable helices *[30]. Both require at least three base pairs in which at least one is a G-C pair. We discovered that semi-stable helices are overwhelmingly very close to other helices in backbone positions. This confirms our conjecture that semi-stable helices interact with other helices on a contiguous strand, i.e., through coaxial stacking [30]. This also suggests a small distance *k *between coaxial stacked helices, consistent with the findings by others [22,25-27]. In this preliminary work, we used *k ≤ *1 as a necessary condition for two neighboring helices to coaxially stack. In our method, coaxial stacking may occur in a two-way junction consisting of two helices sharing both connecting loops or in a multi-way junction joined by multiple helices.

**Definition**. We denote (*X, Y*) to be a coaxial stack between helices *X *and *Y*. Let *L*(*X*) be the set of indexes of nucleotides in the 5'-end base pair region of helix *X*; also let *H*(*X*) be the set of indexes of nucleotides in the 3'-end base pairing region of helix *X*.

1. Coaxial stack (*X, Y*) is *nested *if max *L*(*X*) *<*min *L*(*Y*) and max *H*(*Y*) < min *H*(*X*).

2. Coaxial stack (*X, Y*) is *parallel *if max *H*(*X*) *<*min *L*(*Y*).

In particular, coaxial stacks in two-way junctions are always nested. In multiple-way junctions, coaxial stacks may be either nested or parallel (see Figures 1 and 2). For example, Figure 1 shows two coaxial stacks: a parallel stack between the *D *helix and the anticodon helix, and a nested stack between the *T*_Ψ_*C *helix and the acceptor helix.

The amount of reduced energy, attributed to a coaxial stack, is defined as the free energy contributed from the two stacked base pairs on the interface (see Figure 2). The amount of energy, thus computed via software RNAeval, ranges from -0.9 Kcal/mol to -3.4 Kcal/mol. This is close to the parameter used by Tyagi and Mathews [22].

### Geometric constraints

We applied additional constraints on coaxially stacked helices based on geometric feasibility. This is to consider when two or more coaxial stacks may occur simultaneously, and they all involve some helix. In particular, we identified the following rules to ensure consistency in geometry. Assume helix *X *coaxially stacks with two other helices *Y *and *Z*, then exactly one of the following situations must occur:

1. Stacks (*X, Y*) and (*Z, X*) are nested stacks,

2. Stack (*X, Y*) is nested and stack (*X, Z*) or (*Z, X*) is parallel,

3. Stack (*Y, X*) or (*X, Y*) is parallel and stack (*X, Z*) is nested.

**Figure 3:**
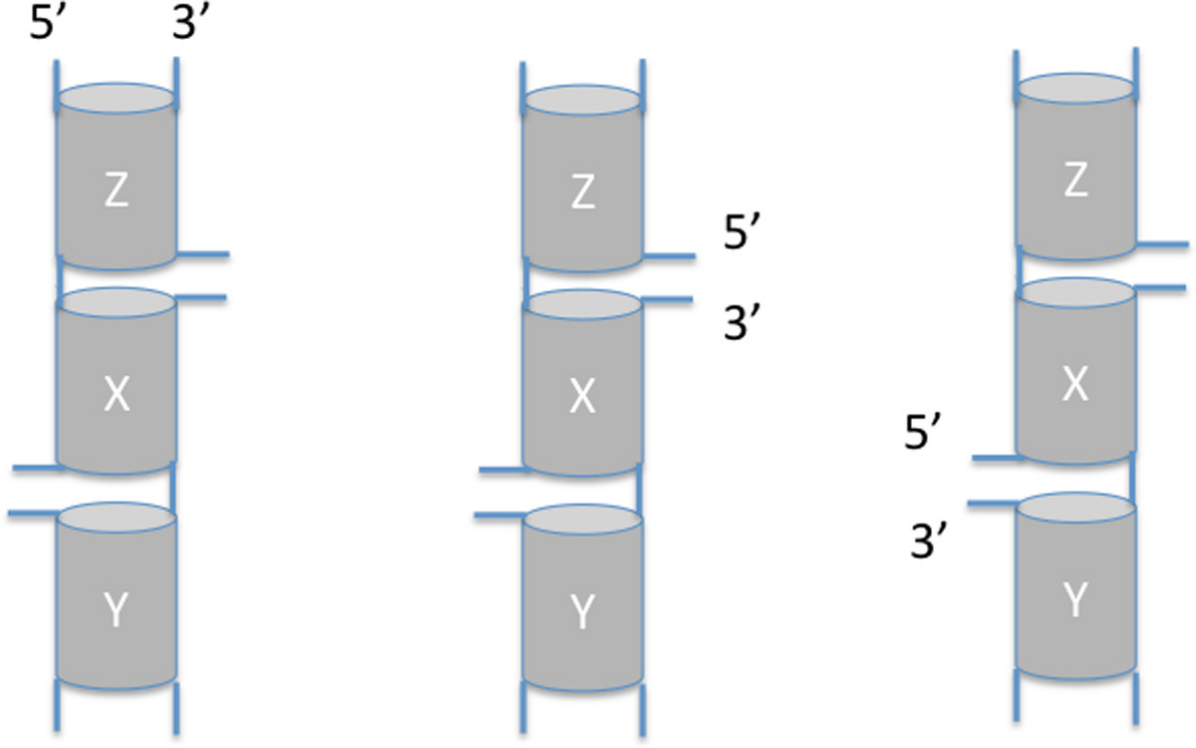
**Compound coaxial stacks**. An illustration for the three general situations of compound coaxial stacks, where 5' and 3' indicate the backbones from the 5' end and to the 3' end of the sequence, respectively. In the left structure, the stacks (*X, Y*) and (*Z, X*) are nested stacks. In the middle structure, the stack (*X, Y*) is nested while (*Z, X*) is parallel. In the right structure, the stack (*X, Y*) is parallel while (*X, Z*) is nested.

Figure 3 illustrates the above compound coaxial stacks 1, 2, and 3, respectively from left to right.

### Algorithm

We developed our method into an algorithm for *ab initio *and simultaneous prediction of secondary structure and coaxial stacks. There are two major phases: *preprocessing *and *prediction*. Given a query RNA sequence, the preprocessing step finds all semi-stable, stable, and ultra-stable helices (see the *Algorithm overview *section below), and also all potential coaxially stacked helix pairs. The computed information is then passed onto the prediction phase, which uses a dynamic programming algorithm in the spirit of Nussinov's algorithm. However, our algorithm is established at helix-level instead of nucleotide-level for the purpose of incorporating coaxial stacking. Since helices cannot be sorted in a linear order, the dynamic programming algorithm design became a non-trivial task.

### Preprocessing of helices

The preprocessing step picks up helix candidates and identifies potential coaxial stacks. A semi-global alignment algorithm is used for searching helix candidates [30]. In a helix candidate, either backbone is allowed to contain at most one unpaired nucleotide. The free energy of helix candidates is measured using RNAeval, a component of the Vienna RNA Package [9,29].

Two helices are recognized as a potential coaxial stack if they share a contiguous single-strand backbone with at most one unpaired nucleotide. Potential coaxial stacks are classified into parallel and nested stacks based on the conditions given in the section above about *Coaxial stacking rules*. The extra energy reduction of a coaxial stacking is computed from the two terminal base pairs of the helices as if they were two contiguous base pairs (see the same section).

### Prediction via dynamic programming

We adopted the idea in Nussinov's algorithm [7] to develop a dynamic programming algorithm for simultaneous prediction of secondary structure and coaxial stacks. Nussinov's algorithm and alike [9,11] use a simple dynamic programming approach, at the nucleotide level, to predict the secondary structure of an RNA. For each subsequence from position *i *to *j*, Nussinov's algorithm computes the substructure with the maximum number of base pairs. In contrast, however, our algorithm works at the helix level in order to incorporate coaxial stacking information. Since helix candidates cannot be sorted in a linear order, the dynamic programming is not straightforward. We addressed this issue by employing partial orderings.

#### Candidates and orderings

A helix consists of two base pairing regions; each region is a contiguous backbone consisting of a number consecutive nucleotides. A helix found by the preprocessing step can be viewed as two base pairing regions. Throughout this section we will refer to candidate regions simply as *candidates*.

On an RNA sequence *x*_1 _... *x_n_*, for each subsequence from position *x_a _*to *x_b _*our algorithm goes through every pair of candidates *i *and *j*, where *i *starts at position *x_a _*and *j *ends at position *x_b_*. The preprocessing may generate several candidates that start at the same position or end at the same position. Therefore, the order in which we visit such overlapping candidates is important to ensure that we always move from smaller subproblems to larger ones. In other words, for the mentioned subsequence, we want to consider the longest candidate *i *and the longest candidate *j *before considering shorter ones. Hence, we assign two different indices to each candidate according to *Starting Position Order *(SPO) and *Ending Position Order *(EPO), i.e., for two candidate regions *r *and *s*, assuming *b*(*r*) gives the starting position of region *r*, and *e*(*r*) gives its ending position, we have:

• *r *≤ _*SPO *_*s*, if *b*(*r*) *< b*(*s*), or if *b*(*r*) = *b*(*s*) &*e*(*r*) *< e*(*s*).

• *r *≤ _*EPO *_*s*, if *e*(*r*) *< e*(*s*), or if *e*(*r*) = *e*(*s*) &*b*(*r*) *< b*(*s*).

If two candidates occupy the exact same region on the sequence, then one of them gets the lower index in a consistent manner throughout the algorithm.

The recurrence relations in our dynamic programming algorithm have the general form *F*(*i, j*), where *F *is a recursive function defined with specific semantic constraints; it gives the maximum score for the optimal substructure (following the mentioned constraints) of the subsequence that starts from the beginning of the candidate with SPO index *i *and ends at the end of the candidate with EPO index *j*. Henceforth, for convenience, *i *always refers to an SPO index, and *j *always refers to an EPO index.

#### Algorithm overview

Similar to Nussinov's algorithm, four different cases can happen when finding the optimal structure of the subsequence spanned from candidate *i *to *j*:

• Region *i *forms a helix (or pairs) with region *j*.

• Region *i *does not participate in the optimal structure.

• Region *j *does not participate in the optimal structure.

• The optimal structure is formed by putting together the optimal substructures of the subsequence from region *i *to region *k*, and of the subsequence from region *k *+ 1 to region *j*, for some *k*.

Our algorithm can recursively generate the following types of topological constructs:

1. An *m*-way junction or a single helix not enclosed by any other helix. Such an *m*-way junction is without a coaxial stacking.

2. An *m*-way junction enclosed by a helix:

(a) an *m*-way junction, without coaxial stacking,

(b) a 2-way junction where the helices coaxially stack,

(c) an *m*-way junction, *m *> 2, with left or right nested coaxial stacking,

(d) an *m*-way junction, *m *> 2, where two of the helices form a parallel stacking.

Each of the three types of helices, defined earlier (see the *Preprocessing *section), contributes differently to building the above topological constructs. A semi-stable helix can appear in the predicted structure only if (a) it coaxially stacks with a stable helix and does not enclose any other helices, or (b) it participates in a 2-way junction and the two helices together are strong enough to act as a stable helix. The only restriction for a stable helix is that it cannot immediately enclose an *m*-way junction. In addition to semi-stable and stable helices, we also have ultra-stable helices. An *ultra-stable *helix has a free energy level lower than -4.6 Kcal/mol and has more than 5 base pairs. For an *m*-way, *m *> 2, junction to exist, it needs to be enclosed by an ultra-stable helix. An exception to this rule is when two helices involved in a 2-way junction, *put together*, are strong enough to act as an ultra-stable helix, then they also can enclose an *m*-way junction. We define different types of recurrences for generating an optimal secondary structure made of the above topological constructs such that the geometric constraints are met and the coaxial stacking rules are followed as well.

• *M *(*i, j*) in which the substructure for the subsequence from region *i *to *j *is not enclosed by any helix; it generates construct 1,

• Functions of the form *M_xy _*(*i, j*) where it is assumed that the structure is enclosed by a helix outside the mentioned subsequence; henceforth, referred to as *the enclosing helix*. Such recurrences do not immediately cause a bifurcation, therefore, they do not immediately enclose an *m*-way, *m *> 2, junction. Instead they may form a helix between *i *and *j*, or ignore *i *and/or *j *in order to move to a smaller subproblem. The subscript *xy *is used to determine the type of the recurrence, and it can be any of

- 2*W: i *and *j *form a helix, and together with the outside enclosing helix they form a 2-way junction that may or may not involve a coaxial stacking.

- @*ϵ*: the left-most helix of the substructure coaxially stacks with the enclosing helix.

- *ϵ*@: the right-most helix of the substructure coaxially stacks with the enclosing helix.

- *ϵ *||: the right-most helix of the substructure forms a parallel coaxial stack with a helix to the right of the subsequence from region *i *to region *j*.

- || *ϵ*: the left-most helix of the substructure forms a parallel coaxial stack with a helix to the left of the subsequence from region *i *to region *j*.

- *ϵϵ*: none of the helices in the substructure coaxially stack with any outside helices.

• Functions of the form $M_{xy}^{B}\left(i,\;j\right)$, where *B *stands for bifurcation, and different cases of the subscript *xy *are defined similar to the ones above. Here the important assumption is that the structure is surrounded by an ultra-stable helix outside the mentioned subsequence. If the outside enclosing helix is not strong enough, but when *put together *with the possible *i, j *helix they can act as an ultra-stable helix, that is also acceptable. In these recurrences, the substructure predicted for the subsequence from region *i *to region *j *will be an *m*-way junction, *m *> 2, that may or may not be enclosed by a possible helix formed by *i *and *j*. The main difference between a function *M_xy _*(*i, j*) and a function $M_{xy}^{B}\left(i,\;j\right)$ is that the latter may *immediately *cause a bifurcation, whereas the former may not.

#### Notation

We use the following notation throughout this section:

• *i *and *i*' are used for referring to indices of candidates in the Starting Position Order (SPO).

• *j *and *j*' are used for referring to indices of candidates in the Ending Position Order (EPO).

• *d *(*a, b*) is the *distance *between candidate regions *a *and *b*. It is the shortest nucleotide distance between the end of candidate *a *and the beginning of candidate *b*, assuming *a *ends before where *b *starts.

• *i *+ 1 is the candidate region after (and possibly overlapping with) *i *in the SPO.

• *j *- 1 is the candidate region before (and possibly overlapping with) *j *in the EPO.

• *s *_≥ *x *_(*i*) is the first non-overlapping successor of *i *in SPO at a distance greater than or equal to *x*.

• *p *_≥ *y *_(*j*) is the first non-overlapping predecessor of *j *in EPO at a distance greater than or equal to *y*.

• $s_{\leq 2}^{\prime }\left(i\right)$ represents any non-overlapping successor of *i *in SPO at distance at most 2 from *i*.

• $p_{\leq 2}^{\prime }\left(j\right)$ represents any non-overlapping predecessor of *j *in EPO at distance at most 2 from *j*.

• *A_ij _*is the weight of any helix (semi-stable, stable, or ultra-stable) formed by *i *and *j*, or - ∞ if no such helix exists.

• *S_ij _*is the weight of a helix *i, j *that is stable or ultra-stable, or - ∞ if no such helix exists.

• *U_ij _*is the weight of a helix *i, j *that is ultra-stable, or - ∞ if no such helix exists.

• *CS *is the reward for a coaxial stacking. Its value depends on the terminal base pairs of the helices involved.

• In rules of the form *F*(*i, j*) = *A_ij _*+ max_*i*',*j*'_{*M*_2*W *_(*i*', *j*')} the requirement is that the helices formed by candidates *i < i*' *< j*' *< j *do not coaxially stack, |*d*(*i, i*') - *d*(*j*', *j*) | ≤ 4, *d*(*i, i*') ≤ 11, *d*(*j*', *j*) ≤ 11, and that *d*(*i, i*') and *d*(*j*', *j*) cannot both be 0.

#### Recurrences

Assuming that the preprocessing step results in *N *candidate regions, the score of the optimal structure for the whole sequence is equal to *M*(1, *N*), where

$$M\left(i,j\right)=\max\left\{\begin{array}{c} S_{ij}\\ M\left(i+1,j\right)\\ M\left(i,j-1\right)\\ \max_{i<k<j}\left\{M\left(i,k\right)+M\left(s_{\geq 1}\left(k\right),j\right)\right\}\\ ST\left(i,j\right) \end{array}\right)$$

The function *ST*(*i, j*) gives the score of the optimal structure for the subsequence from region *i *to region *j*, where *i *and *j *form a helix.

$$ST\left(i,j\right)=\max\left\{\begin{array}{cc} \;A_{ij}+\max_{i^{\prime },j^{\prime }}\left\{M_{2W}\left(i^{\prime },j^{\prime }\right)\right\}, & \;A_{ij}+A_{i^{\prime }j^{\prime }}\;\text{is stable}\\ \;A_{ij}+\max_{i^{\prime },j^{\prime }}\left\{M_{2W}^{B}\left(i^{\prime },j^{\prime }\right)\right\}, & \;A_{ij}+A_{i^{\prime }j^{\prime }}\;\text{is ultra-stable}\\ \; & \;\\ \;A_{ij}+\max_{{s^{\prime }}_{\leq 2}\left(i\right),{p^{\prime }}_{\leq 2}\left(j\right)}\left\{M_{2W}\left({s^{\prime }}_{\leq 2}\left(i\right),\;{p^{\prime }}_{\leq 2}\left(j\right)\right)+CS\right\}, & \;A_{ij}+A_{i^{\prime }j^{\prime }}+CS\;\text{is stable}\\ \;A_{ij}+\max_{{s^{\prime }}_{\leq 2}\left(i\right),{p^{\prime }}_{\leq 2}\left(j\right)}\left\{M_{2W}^{B}\left({s^{\prime }}_{\leq 2}\left(i\right),\;{p^{\prime }}_{\leq 2}\left(j\right)\right)+CS\right\}, & \;A_{ij}+A_{i^{\prime }j^{\prime }}+CS\;\text{is ultra-stable}\\ \; & \;\\ \;U_{ij}+\max_{{s^{\prime }}_{\leq 2}\left(i\right)}\left\{M_{@\epsilon }^{B}\left({s^{\prime }}_{\leq 2}\left(i\right),p_{\geq 2}\left(j\right)\right)+CS\right\} & \;\\ \; & \;\\ \;U_{ij}+\max_{{p^{\prime }}_{\leq 2}\left(j\right)}\left\{M_{\epsilon @}^{B}\left(s_{\geq 2}\left(i\right),{p^{\prime }}_{\leq 2}\left(j\right)\right)+CS\right\} & \;\\ \; & \;\\ \;U_{ij}+\max_{k}\{ & \;\\ \;\;\;\;\max\left\{M_{\epsilon \epsilon }\left(s_{\geq 2}\left(i\right),k\right),M_{\epsilon \epsilon }^{B}\left(s_{\geq 2}\left(i\right),k\right)\right\} & \;\\ \;\;\;\;+\max\left\{M_{\epsilon \epsilon }\left(s_{\geq 1}\left(k\right),p_{\geq 2}\left(j\right)\right),M_{\epsilon \epsilon }^{B}\left(s_{\geq 1}\left(k\right),p_{\geq 2}\left(j\right)\right)\right\} & \;\\ \; & \;\\ \;U_{ij}+\max_{k}\max_{{s^{\prime }}_{\leq 2}\left(k\right)}\{ & \;\\ \;\;\;\;\;\;\max\left\{M_{\epsilon ||}\left(s_{\geq 2}\left(i\right),k\right),M_{\epsilon ||}^{B}\left(s_{\geq 2}\left(i\right),k\right)\right\} & \;\\ \;\;\;\;\;\;+\max\left\{M_{||\epsilon }\left({s^{\prime }}_{\leq 2}\left(k\right),p_{\geq 2}\left(j\right)\right),M_{||\epsilon }^{B}\left({s^{\prime }}_{\leq 2}\left(k\right),p_{\geq 2}\left(j\right)\right)\right\} & \;\\ \;\;\;\;\;\;+CS\} & \;\\ \; \end{array}\right)$$

In the above function, the first case, with *M*_2*W*_, is only allowed when *A_ij _*and *A*_*i*'*j*' _*put together *are strong enough to act as a stable helix. The second case, with $M_{2W}^{B}$, is only allowed when *A_ij _*and *A*_*i*'*j*' _*put together *are strong enough to act as an ultra-stable helix. The situation is similar for cases 3 and 4 where we have 2-way junctions with coaxial stacking. In case 5, with $M_{@\epsilon }^{B}$, helix *U_ij _*forms a left nested coaxial stack with a helix that it encloses, but since the enclosed structure is an *m*-way junction, *m *> 2, helix *U_ij _*has to be ultra stable. Cases 6 is defined and constrained similarly for a right nested coaxial stack. In cases 7 and 8, the helix *U_ij _*encloses an *m*-way junction, *m *> 2, that may either immediately or later on include a coaxial stacking, therefore it has to be ultra-stable. Case 7 results in an *m*-way junction that may later on include a coaxial stacking, whereas case 8 results in an *m*-way junction with a parallel coaxial stacking. Similar constraints are applied to the recurrences below.

The following recurrence is used for generating a helix that does not coaxially stack with any helix outside of the current subsequence.

$$M_{\epsilon \epsilon }\left(i,j\right)=\max\left\{\begin{array}{c} S_{ij}\\ M_{\epsilon \epsilon }\left(i+1,j\right)\\ M_{\epsilon \epsilon }\left(i,j-1\right)\\ ST\left(i,j\right) \end{array}\right)$$

The following recurrence is used for performing bifurcations such that no helix in the substructure coaxially stacks with any helix outside the current subsequence.

$$M_{\epsilon \epsilon }^{B}\left(i,j\right)=\max\left\{\begin{array}{c} \max_{k}\{\\ \;\;\max\left\{M_{\epsilon \epsilon }\left(i,k\right),M_{\epsilon \epsilon }^{B}\left(i,k\right)\right\}\\ \;\;+\max\left\{M_{\epsilon \epsilon }\left(s_{\geq 1}\left(k\right),j\right),M_{\epsilon \epsilon }^{B}\left(s_{\geq 1}\left(k\right),j\right)\right\}\}\\ \max_{k}\max_{{s^{\prime }}_{\leq 2}\left(k\right)}\{\\ \;\;\;\;\max\left\{M_{\epsilon ∥}\left(i,k\right),M_{\epsilon ∥}^{B}\left(i,k\right)\right\}\\ \;\;\;\;+\max\left\{M_{∥\epsilon }\left({s^{\prime }}_{\leq 2}\left(k\right),j\right),M_{∥\epsilon }^{B}\left({s^{\prime }}_{\leq 2}\left(k\right),j\right)\right\}\\ \;\;\;\;+CS\} \end{array}\right)$$

The following recurrence is used for the case that helix *A_ik _*forms a left nested coaxial stack with a helix that encloses the subsequence from region *i *to region *j*.

$$M_{@\epsilon }\left(i,j\right)=\max\;\left\{\max\left\{A_{ik},ST\left(i,k\right)\right\}\max\left\{M_{\epsilon \epsilon }\left(s_{\geq 1}\left(k\right),j\right),M_{\epsilon \epsilon }^{B}\left(s_{\geq 1}\left(k\right),j\right)\right\}\right\}$$

Similarly, the following recurrence is used for the case that helix *A_kj _*forms a right nested coaxial stack with a helix that encloses the subsequence from region *i *to region *j*.

$$M_{\epsilon @}^{B}\left(i,\;j\right)=\underset{k}{\max}\left\{\max\left\{M_{\epsilon \epsilon }\left(i,p_{\geq 1}\left(k\right)\right),M_{\epsilon \epsilon }^{B}\left(i,p_{\geq 1}\left(k\right)\right)\right\}+\max\left\{A_{kj},ST\left(k,\;j\right)\right\}\right\}$$

The following recurrence is used for the case that helix *A_ij _*forms a 2-way junction with an outside enclosing helix, and it may also form a 2-way junction with a helix in the substructure it encloses.

$$M_{2W}\left(i,j\right)=\max\left\{\begin{array}{cc} \;A_{ij} & \;\\ \;A_{ij}+\max_{i^{\prime },j^{\prime }}\left\{M_{2W}\left(i^{\prime },j^{\prime }\right)\right\}, & \;A_{ij}+A_{i^{\prime }j^{\prime }}\;\text{is stable}\\ \;A_{ij}+\max_{i^{\prime },j^{\prime }}\left\{M_{2W}^{B}\left(i^{\prime },j^{\prime }\right)\right\}, & \;A_{ij}+A_{i^{\prime }j^{\prime }}\;\text{is ultra-stable}\\ \; & \;\\ \;A_{ij}+\max_{{s^{\prime }}_{\leq 2}\left(i\right),{p^{\prime }}_{\leq 2}\left(j\right)}\left\{M_{2W}\left({s^{\prime }}_{\leq 2}\left(i\right),{p^{\prime }}_{\leq 2}\left(j\right)\right)+CS\right\}, & \;A_{ij}+A_{i^{\prime }\;j^{\prime }}+CS\;\text{is stable}\\ \;A_{ij}+\max_{{s^{\prime }}_{\leq 2}\left(i\right),{p^{\prime }}_{\leq 2}\left(j\right)}\left\{M_{2W}^{B}\left({s^{\prime }}_{\leq 2}\left(i\right),{p^{\prime }}_{\leq 2}\left(j\right)\right)+CS\right\}, & \;A_{ij}+A_{i^{\prime }\;j^{\prime }}+CS\;\text{is ultra-stable}\\ \; \end{array}\right)$$

The following recurrence is used for the case that helix *A_ij _*forms a 2-way junction with an outside enclosing helix, but unlike the case for *M*_2*W*_, it immediately encloses an *m*-way junction, where *m *> 2.

$$M_{2W}^{B}\left(i,j\right)=\max\left\{\begin{array}{c} A_{ij}+\max_{{s^{\prime }}_{\leq 2}\left(i\right)}\left\{M_{@\epsilon }^{B}\left({s^{\prime }}_{\leq 2}\left(i\right),p_{\geq 2}\left(j\right)\right)+CS\right\}\\ A_{ij}+\max_{{p^{\prime }}_{\leq 2}\left(j\right)}\left\{M_{\epsilon @}^{B}\left(s_{\geq 2}\left(i\right),{p^{\prime }}_{\leq 2}\left(j\right)\right)+CS\right\}\\ A_{ij}+\max_{k}\{\\ \;\;\max\left\{M_{\epsilon \epsilon }\left(s_{\geq 2}\left(i\right),k\right),M_{\epsilon \epsilon }^{B}\left(s_{\geq 2}\left(i\right),k\right)\right\}\\ \;\;+\max\left\{M_{\epsilon \epsilon }\left(s_{\geq 1}\left(k\right),p_{\leq 2}\left(j\right)\right),M_{\epsilon \epsilon }^{B}\left(s_{\geq 1}\left(k\right),p_{\leq 2}\left(j\right)\right)\right\}\}\\ A_{ij}+\max_{k}\max_{{s^{\prime }}_{\leq 2}\left(k\right)}\{\\ \;\;\;\;\max\left\{M_{\epsilon ∥}\left(s_{\geq 2}\left(i\right),k\right),M_{\epsilon ∥}^{B}\left(s_{\geq 2}\left(i\right),k\right)\right\}\\ \;\;\;\;+\max\left\{M_{∥\epsilon }\left({s^{\prime }}_{\leq 2}\left(k\right),p_{\leq 2}\left(j\right)\right),M_{∥\epsilon }^{B}\left({s^{\prime }}_{\leq 2}\left(k\right),p_{\leq 2}\left(j\right)\right)\right\}\\ \;\;\;\;+\;CS\} \end{array}\right)$$

The following recurrence is used for generating a helix with the assumption that it forms a parallel coaxial stacking with a helix to the right of the subsequence from region *i *to region *j*.

$$M_{\epsilon ∥}\left(i,j\right)=\max\left\{\begin{array}{c} \;A_{ij}\\ \;M_{\epsilon ||}\left(i+1,j\right)\\ \;ST\;\left(i,j\right) \end{array}\right)$$

The following recurrence is used for performing bifurcations such that the right-most helix of the resulting substructure forms a parallel coaxial stacking with a helix to the right of the subsequence from region *i *to region *j*.

$$M_{\epsilon ∥}^{B}\left(i,j\right)=\max\left\{\begin{array}{c} \;M_{\epsilon ||}^{B}\left(i+1,j\right)\\ \;\max_{k}\{\\ \;\;\max\left\{M_{\epsilon \epsilon }\left(i,k\right),M_{\epsilon \epsilon }^{B}\left(i,k\right)\right\}\\ \;\;+\max\left\{M_{\epsilon ||}\left(s_{\geq 1}\left(k\right),j\right),M_{\epsilon ||}^{B}\left(s_{\geq 1}\left(k\right),j\right)\right\}\} \end{array}\right)$$

Similarly we define the recurrences *M*_||*ϵ *_and $M_{∥\epsilon }^{B}$ for the cases that the left-most helix of the resulting substructure forms a parallel coaxial stack with a helix to left of the subsequence from region *i *to region *j*.

$$M_{||\epsilon }\left(i,j\right)=\max\left\{\begin{array}{c} A_{ij}\\ M_{||\epsilon }\left(i,j-1\right)\\ ST\;\left(i,j\right) \end{array}\right)$$

$$M_{||\epsilon }^{B}\left(i,j\right)=\max\left\{\begin{array}{c} \;M_{||\epsilon }^{B}\left(i,j-1\right)\\ \;\max_{k}\{\\ \;\;\max\left\{M_{||\epsilon }\left(i,k\right),M_{||\epsilon }^{B}\left(i,k\right)\right\}\\ \;\;+\max\left\{M_{\epsilon \epsilon }\left(s_{\geq 1}\left(k\right),j\right),M_{\epsilon \epsilon }^{B}\left(s_{\geq 1}\left(k\right),j\right)\right\}\} \end{array}\right)$$

## Abbreviations

**SPO**: Starting Position Order; **EPO**: Ending Position Order.

## Competing interests

The authors declare that they have no competing interests.

## Authors' contributions

PS designed and implemented the prediction algorithm. In addition to contributing to drafting this manuscript, he was also in charge of acquiring data, testing, and analysing the results. YW designed and implemented the pre-processing algorithm. He also helped with data acquisition and result analysis. RM provided the biological insight, and also contributed to data acquisition and results analysis. LC conceived the overall model and algorithm and drafted the manuscript. All authors read and approved the manuscript.
